# Understanding Reduced Rotavirus Vaccine Efficacy in Low Socio-Economic Settings

**DOI:** 10.1371/journal.pone.0041720

**Published:** 2012-08-06

**Authors:** Benjamin A. Lopman, Virginia E. Pitzer, Rajiv Sarkar, Beryl Gladstone, Manish Patel, John Glasser, Manoj Gambhir, Christina Atchison, Bryan T. Grenfell, W. John Edmunds, Gagandeep Kang, Umesh D. Parashar

**Affiliations:** 1 National Center for Immunization and Respiratory Diseases, Centers for Disease Control and Prevention, Atlanta, Georgia, United States of America; 2 Department of Ecology and Evolutionary Biology, Princeton University, Princeton, New Jersey, United States of America; 3 Fogarty International Center, National Institutes of Health, Bethesda, Maryland, United States of America; 4 Department of Gastrointestinal Sciences, Christian Medical College, Vellore, Tamil Nadu, India; 5 MRC Center for Outbreaks Analysis and Modelling, Imperial College London, London, United Kingdom; 6 Department of Infectious Disease Epidemiology, London School of Hygiene and Tropical Medicine, London, United Kingdom; National Institutes of Health, United States of America

## Abstract

**Introduction:**

Rotavirus vaccine efficacy ranges from >90% in high socio-economic settings (SES) to 50% in low SES. With the imminent introduction of rotavirus vaccine in low SES countries, understanding reasons for reduced efficacy in these settings could identify strategies to improve vaccine performance.

**Methods:**

We developed a mathematical model to predict rotavirus vaccine efficacy in high, middle and low SES based on data specific for each setting on incidence, protection conferred by natural infection and immune response to vaccination. We then examined factors affecting efficacy.

**Results:**

Vaccination was predicted to prevent 93%, 86% and 51% of severe rotavirus gastroenteritis in high, middle and low SES, respectively. Also predicted was that vaccines are most effective against severe disease and efficacy declines with age in low but not high SES. Reduced immunogenicity of vaccination and reduced protection conferred by natural infection are the main factors that compromise efficacy in low SES.

**Discussion:**

The continued risk of severe disease in non-primary natural infections in low SES is a key factor underpinning reduced efficacy of rotavirus vaccines. Predicted efficacy was remarkably consistent with observed clinical trial results from different SES, validating the model. The phenomenon of reduced vaccine efficacy can be predicted by intrinsic immunological and epidemiological factors of low SES populations. Modifying aspects of the vaccine (e.g. improving immunogenicity in low SES) and vaccination program (e.g. additional doses) may bring improvements.

## Introduction

Diarrhea causes an estimated 1.3 million childhood deaths per year, of which approximately one-third are a result of rotavirus infections. [1], [2] The WHO in 2009 issued a global recommendation for the inclusion of rotavirus vaccines into routine immunization programs, based on results from successful clinical trials from Asia and Africa. [3], [4], [5], [6] These vaccines have great potential to prevent the severe morbidity and mortality from rotavirus, but studies consistently demonstrate a gradient of reduced efficacy in low socio-economic settings (SES) where the burden of severe rotavirus disease, particularly mortality, is greatest. [7] Clinical trials and observational studies of oral rotavirus vaccines performed in infants in high income settings demonstrated vaccine efficacy (VE) exceeding 90%. [8], [9], [10], [11], [12] In middle income settings of Latin America, South Africa and Vietnam,VE ranged from 72 to 83%, [5], [13] while in low income settings in Asia and Africa, VE ranged from 39 to 49%. [4], [5], [6] These same patterns are apparent for both currently licensed vaccines (single-strain Rotarix® (GlaxoSmithKline Biologicals) and pentavalent Rotateq® (Merck & Co) referred to henceforth as RV1 and RV5, respectively). Understanding the biological basis for this poorer performance may be crucial for maximizing the impact of current vaccines and for guiding the development of new ones.

With all existing live oral vaccines against enteric infections (including typhoid, cholera and oral polio), the immune response and efficacy are diminished amongst certain populations living in developing countries. [4], [5], [6], [14], [15], [16] While the exact reasons for this phenomenon are unclear, a range of hypotheses has been proposed. These can be broadly categorized as (1) factors leading to a poor immune response to natural infection, (2) reduced immunogenicity of the vaccines, and (3) very high incidence rate of infection that overwhelms immunity from vaccination. Overgrowth of bacteria in the small bowel (‘tropical enteropathy’), [17] concomitant infections including helminths, [18] micronutrient deficiency [19] and nutritional status are specific factors that have all been associated with diminished immune response to live oral vaccination, and would also affect immunity to natural infection. Furthermore, the greater diversity of rotavirus strains circulating in many developing countries may lead to weaker natural and vaccine-derived immunity. [20] In very young infants in low SES, greater levels of maternal antibody acquired trans-placentally or from breast milk may serve to neutralize vaccine virus so as to reduce replication and antigen load and thereby decrease the immunogenicity. [21] Identifying whether these factors can explain the reduced vaccine efficacy observed in low SES, and which factors are most important, can help to prioritize future research aimed at improving vaccine performance.

We aimed to interpret rotavirus clinical trial and field effectiveness observations in a range of SES using a mathematical model and to illustrate how these different factors may contribute to reduced efficacy of vaccination. We used a dynamic mathematical model that incorporates age structure and the natural history of rotavirus infection and immunity. Three scenarios were constructed to reflect the protection conferred by natural infection and vaccination, immunogenicity of vaccines, and disease incidence in high, middle, and low SES. Based on our findings, we identify and discuss potential strategies to improve the performance of rotavirus vaccines in low SES.

## Methods

### Model

We developed and analyzed a deterministic mathematical model of rotavirus transmission and disease (see Figure S1 and Equations S1).; [22] The model is dynamic and incorporates age structure and the natural history of rotavirus infection and immunity. Individuals are born into a class where they are protected by maternal antibody. Immunity wanes at a constant rate, after which individuals move into the first susceptible class. Immunity to rotavirus infection is complex and incomplete, reflecting what is known about the natural history of rotavirus; we included four susceptible (*S*_1_–*S*_4_) and infected (*I*_1_–*I*_4_) classes. Individuals move from *S*_n_ to *I*_n_ at an age-specific force of infection (*λ_i_*). Once infected, a proportion (*ξ_n_*) develops symptomatic rotavirus gastroenteritis (RV-GE), *φ_n_* of which are severe (defined as a score ≥11 on the Vesikari scale). [23] These proportions vary depending on the order of infection (primary, secondary, tertiary or quaternary) and SES. Following infection, a proportion *α_n_* enters the next susceptible class, while 1-*α_n_* develops long-term immunity. Only symptomatic individuals are assumed to contribute to transmission, and all episodes are assumed to be of the same duration (1/*γ*_n_ = 7 days).

### Scenarios

Three scenarios were constructed to reflect the protection conferred by natural infection and vaccination, immunogenicity of vaccines, and disease incidence in high, middle and low SES.

#### Protection from natural infection and vaccination

The protection conferred by natural infection has been estimated by Velazquez et al. [24] in Mexico and more recently by Gladstone et al. [25] in South India. Both studies demonstrated increased protection against infection with each subsequent exposure to rotavirus. However, while the proportion of infected infants with symptomatic disease and the proportion of them with severe disease decreased following each subsequent infection in Mexico, no clear pattern was observed in South India. No studies of this type have been performed in higher income settings, so we used the Mexico data to represent natural history in high and middle SES and the South India data to represent low SES.

Rotavirus immunization is by live oral vaccination, and mechanistically is believed to mimic immunity from natural infection. We assumed that each dose of vaccine acts like a single infection, without causing symptomatic disease. Two doses of vaccine, given at 2 and 4 months of age, were assumed to confer protection equivalent to primary and secondary infection.

#### Immunogenicity of vaccines

We assumed that only a proportion of children who received a given dose of vaccine are protected, [26] as determined by the proportion who seroconvert. The rates of seroconversion for high, middle, and low SES were based on a literature review of 24 immunogenicity studies of RV1, where seroconversion was defined as GMC >20 U/mL. [27] We explored the plausible range in predicted VE that may result from variation in immunogenicity by also running the model at the highest and lowest seroconversion rate reported from studies from each SES. In this framework, vaccination is “all-or nothing”, in the sense that a dose either confers protection or not. However, the two doses mimic first and second natural infections in the sense that vaccination, like prior infection, mitigates against disease: a greater degree of mitigation is associated with each consecutive vaccine dose (as is the case for each natural infection).

#### Incidence

Based on the natural history data, the high, middle, and low SES models were fit by adjusting *q* (the infectivity parameter) to age-specific incidence data from cohort studies in England [28], Mexico [24], and India [25] to represent the three settings (Figures S2). The best-fit models were determined by minimizing the root-mean-square deviation (RMSD) in Berkeley-Madonna (Berkeley, CA, USA). In all settings, it was assumed that the population size was stable over the period of the study (births equal to deaths), but that birth and death rates were inversely associated with SES (life expectancy of 80, 75 and 60 years corresponding to birth rates of 12.5, 13.3, and 16.7 live births per 1,000 per year in high, middle and low SES, respectively). We also investigated a higher birthrate of 25.0 births per 1,000 per year in low SES, which is more realistic for India. As a simplifying assumption, we assumed that all deaths occurred in the adult age class (25 years and older). Levels of maternal immunity were assumed to be greater in low SES (Table 1). [21], [27], [29].

**Table 1:** Model parameters.

			High	Middle	Low	Symbol
Duration of maternal immunity (weeks) [21], [29]			13	13	26	1/µ
Life expectancy (years)			80	75	60	1*/d*
Duration of infectiousness (days) [23]			7	7	7	1/*γ*
Relative risk of infection following						
	First infection		0.62	0.62	0.61	*α*_1_
	Second infection		0.37	0.37	0.48	*α*_2_
	Third infection		0.37	0.37	0.33	*α*_3_
Proportion of infections with any GE (severe GE)						
	First infection		0.47 (0.28)	0.47 (0.28)	0.30 (0.17)	*ξ*_1_ (*φ*_1_)
	Second infection		0.24 (0.19)	0.24 (0.19)	0.28 (0.23)	*ξ*_2_ (*φ*_2_)
	Third infection		0.32 (0)	0.32 (0)	0.18 (0.24)	*ξ*_3_ (*φ*_3_)
	Fourth infection		0.20 (0)	0.20 (0)	0.21 (0.18)	*ξ*_4_ (*φ*_4_)
	*Source*		[*24*]	[*24*]	[25]	
Proportion who seroconvert to vaccination [27]			0.86 (0.67 to 0.98)	0.74 (0.63 to 0.86)	0.63 (0.58 to 0.67)	*c*
Infectivity parameter*		*Fitted*	0.235781	0.604687	2.25781	*q*

* The infectivity parameter *q* represents the proportion of infectious contacts (i.e. when a susceptible and infectious individual come into contact) that result in transmission, multiplied by a constant factor by which the contact rate is assumed to scale across settings.

The best fit models, with SES-specific parameters, were then used to analyze vaccine efficacy as described below.

### Efficacy

Efficacy was defined as the proportion of cases prevented per vaccinated child. In this framework, vaccine efficacy is complex: it is a function of age-specific incidence by order of infection (primary, secondary, etc), the proportion symptomatic (*δ_n_*) and severe (*φ_n_*) at the *n* ^th^ infection, as well as the ‘take’ of the vaccine. For these reasons, it was not possible to solve analytically for the age-specific efficacy. In order to isolate the direct effect of vaccination, we fixed the age- and setting-specific force of infection to the equilibrium pre-vaccination value derived from the model fitting. After fitting the dynamic model, the force of infection was fixed using the parameters of the best fit model, and the model was run to endemic. The model was run for 60 years to achieve endemic equilibrium (which is achieved quickly when the force of infection is fixed) and stable age structure before introducing vaccination. Efficacy was calculated as one minus the incidence at equilibrium in a fully vaccinated cohort divided by the incidence in an unvaccinated cohort. This approach is used to compare incidence in vaccinated and unvaccinated groups without allowing vaccination to affect the transmission dynamics, representing a trial scenario.

Efficacy in the 6 to 23 month age group is presented as the main outcome measure to facilitate comparison with the age group that was primarily followed for clinical outcomes in vaccine trials. We estimated absolute reductions as cases prevented per cohort of 1000 vaccinated children.

The incremental effect of the immune response to natural infection and vaccination, the immunogenicity of vaccines, and the underlying incidence rate on efficacy was determined by changing each parameter in a stepwise fashion. Starting with the baseline model with low SES parameters, we changed the proportion who seroconvert (*c*) from 0.63 (low) to 0.74 (middle) then to 0.86 (high); next we changed the natural history parameters (*α*_x_, *ξ*_x_ and *φ*_x_) from the values from Velazquez et al [24] to Gladstone et al [25]; finally, the force of infection was changed by altering *q* from *q_low_* to *q_mid_* to *q_high_* (Table 1). In addition, we also considered the potential value of a third dose of vaccine given at 6 months of age in low SES, assuming that protection from a third dose is the same as a third natural infection.

## Results

**Figure 1:**
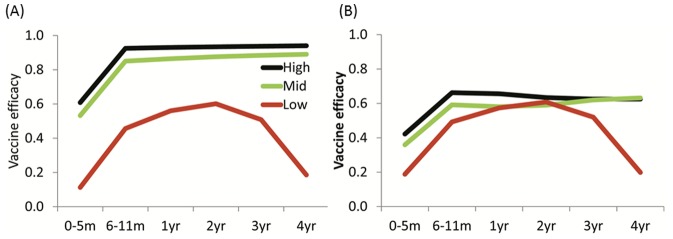
Predicted age-specific efficacy on severe RV-GE and all RV-GE. A) Vaccine efficacy: Severe RV-GE. B) Vaccine efficacy: All RV-GE.

In the 6 to 23 month age group, the model generated vaccine efficacy (VE) estimates against severe RV-GE of 93%, 86% and 51% in high, middle and low SES, respectively (Table 2). Under the higher birth rate scenario (25 per 1000 per year) in low SES, our estimate of VE was marginally reduced (by 2%). When considering the range of seroconversion values in the literature, we predicted VE to range from 79% to 99% in high SES, 77% to 93% in middle SES and 47% to 53% in low SES. Against all RV-GE, VE was 66%, 58% and 53%, respectively. VE decreased in low SES in 3 year olds and was negligible in 4 year olds. In contrast, VE did not decrease amongst older age groups in middle or high income settings (Figure 1).

**Table 2:** Predicted vaccine efficacy and severe cases prevented per 1000 vaccinated children in high-, middle- and low-socio economic settings.

	Vaccine efficacy		Cases prevented	
	0 to 4 yrs	6 m to 23 m	0 to 4 yrs	6 m to 23 m
Severe RV-GE				
High	0.91	0.93	139	63
Middle	0.81	0.86	202	133
Low	0.41	0.51	89	71
All RV-GE				
High	0.63	0.66	392	167
Middle	0.57	0.58	660	374
Low	0.44	0.53	537	410

* Results are presented in these two age groups to facilitate comparison with clinical trials (6–23 m) and population impact assessments (0 to 4 yrs).

From 6 to 23 months of age, 71 cases of severe RV-GE per year were estimated to be prevented for every 1000 vaccinated children in low SES, while 63 cases per 1000 vaccinated were prevented in high SES (Table 2). However, in older age groups, this pattern was reversed, with larger gains in higher income settings. This is a function of both the younger age distribution of rotavirus and lower efficacy of the vaccine in low SES. By age 5 years, vaccination of 1000 children would prevent 139, 202, and 89 severe cases in high, middle, and low SES, respectively.

**Figure 2:**
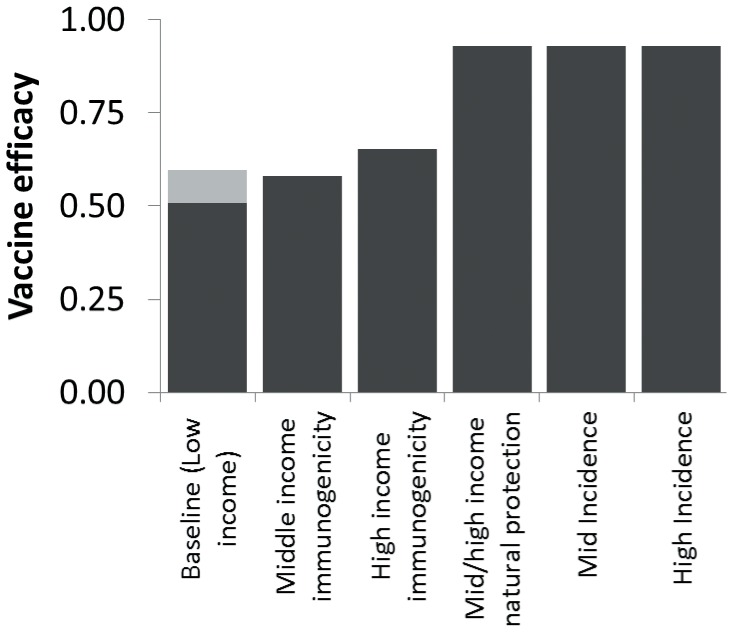
Predicted vaccine efficacy on severe rotavirus gastroenteritis incidence in 6 to 23 month-olds. Step-wise influence of improving the underlying natural history of protection, immunogenicity of vaccines, and baseline disease incidence. The gray shaded area on the baseline bar indicates the potential incremental increase in VE from a third dose given at 6 months of age.

Starting from a baseline of 51% efficacy among 6 to 23 month-olds in low SES, efficacy was projected to improve to 58% and 65%, respectively, if immunogenicity of vaccination was increased to levels from middle and high income countries (Figure 2 and Figure S3). The rest of the gap in efficacy (to 93% in high income settings) was a result of differences in the protection conferred by natural infection. Underlying incidence had no long-term impact on vaccine efficacy. A third dose of vaccine given at 6 months of age was predicted to increase efficacy from 51 to 60%.

**Figure 3:**
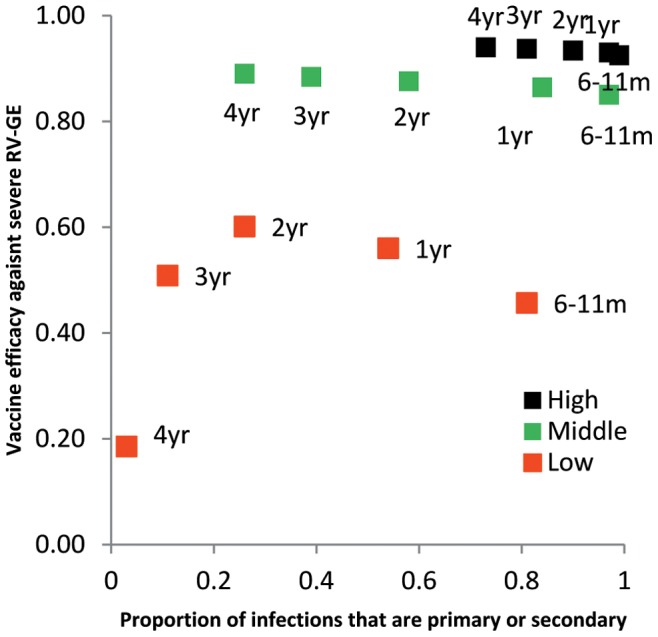
Relationship between vaccine efficacy against severe RV-GE and the proportion of infections that are primary or secondary, by age group. In high (black points) and middle (green points) SES VE remains stable across age groups, despite the fact that the proportions of infections decrease with age, because in these settings all severe disease is confined to the first two infections. In low SES, VE falls as the proportion of infections that are primary or secondary decreases because severe disease continues to occur in subsequent infections.

Figure 3 illustrates the relationship between age-specific vaccine efficacy estimates and the proportion of infections that were either primary or secondary (as projected by the model). In high and middle SES, VE remained steady as the proportion of infections that were primary or secondary decreased with age. In low SES, VE fell with this proportion.

## Discussion

This modeling study demonstrates that the phenomenon of reduced rotavirus vaccine efficacy in low SES can be explained by intrinsic immunological and epidemiological factors. While natural rotavirus infections protect against subsequent infections across a range of SES, a key difference is that, unlike in middle and high SES, in low SES the proportion of infections that result in symptomatic disease does not rapidly decline with each subsequent infection; a similar proportion of primary or secondary infections result in severe rotavirus disease when compared with tertiary and subsequent infections. Thus, if vaccination mimics primary and/or secondary natural infection(s), subsequent infections and illnesses will not be adequately protected against, resulting in lower VE and shorter apparent duration of protection in low SES where subsequent infections occur at a high rate and continue to be severe.

A number of model VE outputs, predicted based on the natural history and immunogenicity data, are consistent with clinical trial findings, which is reassuring regarding the validity of our assumptions. First and foremost, the model predicts higher VE with increasing SES. Efficacy against severe RV-GE was similar in our model (93%) as it was for RV1 and RV5 in clinical trials and observational studied in high SES, where VE of greater than 90% was observed. [30], [31] Similar effectiveness estimates have been found in post-marketing surveillance studies. [8], [32], [33], [34] Our estimate of 86% efficacy against severe RV-GE in middle income countries is again consistent with trials across Latin America (83%), in South Africa (77%), and Vietnam (72%), and observational studies of effectiveness in El Salvador (76%) and Brazil (76%). [4], [5], [13], [35], [36] Our VE estimate of 51% in low SES populations approximates the VE of 49% in Malawi, 43% in Bangladesh, and 39% observed in three sub-Saharan African countries. [4], [5], [6] Our main results relied on the mean seroconversion rates by SES. However, in limited sensitivity analysis, we demonstrated that the range of seroconversion rates observed in immunogenicity studies may also explain some of the variation in VE from clinical trials.

Secondly, the model projects VE against severe RV-GE to be approximately 25% greater than against all RV-GE in middle and high SES. This is remarkably consistent with trial data from high SES. [31] However, the model does not predict this differential efficacy in low SES because the proportion of cases that are severe is similar with each subsequent infection. The limited data from these settings indicates that the gap between severe and all RV-GE VE may be smaller. [4], [5], [6] If future data on all-severity RV-GE in low SES support this prediction, it will give further support to the notion that the mechanism of vaccine action is to mimic natural infection, as well as helping to understand the potential impact of the vaccine on transmission in these settings.

Third, the model predicts and provides an explanation for what appears to be ‘waning’ of VE in low SES but not high SES populations, which has been observed in the clinical trial data. In 3 African settings, efficacy of RV5 against severe RV-GE was estimated to be 64% in the first year of life, falling to 20% in the second year [6]; smaller, but nonetheless important declines have been observed in El Salvador, Nicaragua and Brazil, [35], [37], [38] though it should be noted that studies have generally not been powered to estimate effectiveness in second year of life and beyond. The model does not fully capture how quickly VE falls; in clinical trials, VE declined by the second year of life and in the model, it fell in the third. However, waning – traditionally defined as loss of immunity over time – is not an influential feature of the model (as waning occurs on a scale of >40 years). Even without loss of immunity, VE as measured by clinical trials or cohort studies, can, in some circumstances, fall with increasing age. If vaccination reduces the force of infection and provides only partial protection for vaccinated individuals, proportionally more cases will occur in older age groups amongst vaccinated than unvaccinated individuals. [39] Still, a better model fit could perhaps be achieved by explicitly incorporating loss of immunity, in effect representing local versus systemic immunity. However, the data needed to parameterize such a model for a range of SES are not available.

Because immunity to rotavirus is incrementally-acquired, our model proposes a different mechanism to explain reduced VE in older children. In the model, each dose of the vaccine mimics a natural infection. With a two-dose course, all children who respond to vaccination in higher SES are protected against severe disease as all severe disease is thought to occur as a result of the first two infections. In low SES, the mechanism of the vaccine is the same and higher order infections do confer additional protection against infection [24], but severe disease continues to occur in third, fourth and subsequent infections. These higher order infections make up a larger proportion of infections as children age, and are not protected against by vaccination, so in older children VE appears to ‘wane’. This is an important observation in that it suggests that additional vaccinations, for example a dose given with measles vaccination in the EPI schedule at 9 or 12 months, may improve performance among children in low SES, although the level of protection conferred by such schedules needs to be clinically evaluated.

We have assumed that the immune response to natural infection and vaccination, immunogenicity of vaccines, and background rotavirus incidence are independent factors, and this may be an important limitation. For example, part of the reason that live oral vaccines may be less effective is due to concomitant infections of the gut, as had been posited for OPV. [16] Rotavirus and other concomitant infections will both be more common in low SES/high incidence settings, where vaccines also appear to be less immunogenic. [27] As we have demonstrated, background rotavirus incidence itself may affect the impact of the vaccine program, but not VE directly; concomitant infections could still explain lower VE by interfering with both the immune response to vaccination and immunogenicity of vaccines.

We have also assumed that the severity of disease is dependent on the number of previous infections (and decreases with each subsequent one). However, it remains possible that severity is age-dependent. If, for instance, under 1 year-olds are more susceptible to severe disease regardless of the number of previous infections, just delaying age at infection will reduce severe disease. Age and number of previous infections may confound each other, but due to limitations in available data it is difficult to disentangle these factors. It is important to note that the younger age distribution of infection in low SES may at least partly explain the discrepancy in natural immunity between mid/high and low SES. Research directed at this issue may help to elucidate the extent to which simply delaying time to infection could result in a reduction in severe disease.

We are not aware of any robust data on mixing patterns and contact structures for either middle or low income settings, so we assumed mixing was proportional to age-specific patterns for Great Britain from a large European study. [40] These data are unlikely to represent mixing patterns in either Mexico or India. We account for this, at least in part, by allowing the parameter *q* to vary. *q* represents the probability of transmission given a contact between a susceptible and infectious person. However, *q* may also be interpreted as a composite of infectiousness and frequency of contact, so a higher *q* ultimately represents a higher force of infection, which could result from greater infectiousness or more frequent contacts. Further studies are needed to elucidate mixing patterns for middle and low income settings.

Underpinning our results are the findings from the Indian natural history study that severe disease continues to occur in third and subsequent infections, whereas in Mexico severe cases are principally restricted to primary infection. A host of reasons for this discrepancy are possible. Exposure to higher doses of virus may occur in low SES, which could overcome immunity from previous infections. Greater strain diversity and more limited cross-protective immunity may also play a role. In addition to the mechanism we have modeled, immunity from both natural infection and vaccination may wane (in the “traditional” sense), whereby protective antibody is lost over time in children in low SES.

We have taken data from the UK, Mexico and India to be a general reference for the diverse range of epidemiological and demographic profiles of high, middle and low SES worldwide. Clearly, this is a simplification as factors such as crowding and underlying rates of diarrhea differ both between and within countries such as India, resulting in lower VE. [16] Despite this simplification, we were able to match many of the observations of rotavirus vaccine clinical trials, suggesting that this framework is a useful tool for understanding some of the variation in VE across populations.

Our results help identify potential strategies to improve the performance of rotavirus vaccines in low SES. The immunogenicity of vaccines is likely to be the most directly modifiable of the factors investigated. Some vaccines in development, such as the neonatal 116E strain currently being trialed in India, may be more immunogenic than currently licensed vaccines. [41] A second strategy to improve immunogenicity may be directed at the host. Delaying administration of vaccine from the current 6 and 10 weeks schedule (with RV1) to 10 and 14 weeks [5] may also be an effective strategy to improve immunogenicity by allowing maternal antibody to wane for another four weeks [5], though this approach would have to be weighed against the risks of early natural infections and the potential risk of intussusception with later vaccination. A schedule with 3 doses of RV1 given at 6, 10, and 14 weeks might offer the most practical and programmatically feasible option, given that regulatory and economic considerations are satisfactorily addressed. We estimate that a third dose of vaccine may improve VE by approximately 9% in low SES. This finding is based on the assumption that a third dose of vaccine is as immunogenic as the first two doses, though there are little empirical data presently available to support this. [5] Studies are needed to characterize the immune response and protection conferred by a third dose of vaccine, and to specifically determine if immunogenicity is compromised for doses administered at very young ages as a result of interference from maternal antibody.

In summary, this study demonstrates that even in their current sub-optimal state, rotavirus vaccines have the potential to substantially reduce severe diarrheal disease in very young children in low SES. By identifying and quantifying factors resulting in poorer vaccine performance in these settings, we are able to propose both a mechanism by which vaccination provides protection and an estimate of what can realistically be achieved. Modifying aspects of the vaccine (e.g. improving immunogenicity in low SES populations) or vaccination program (e.g. additional doses) may bring improvements, but in order to fully realize the benefits of the vaccine, interventions targeted at the host and the broader epidemiology of disease may be required.

## Supporting Information

Equations S1 **Model equations and additional description**
(PDF)

Figure S1 **Model structure.** Figure (A) shows the original transmission model by Atchison et al and (B) illustrates how vaccination was included in the current analysis.
(PDF)

Figure S2 **Model-fitted and observed age-specific incidence of all RV-GE.** Observed incidence rates (per 1000 child-years) are in shaded bars; fitted rates are shown in lines. Incidence data were not available across the age range from middle or low SES and in no settings was the age distribution available from the age-intervals of interest in this modeling study, so the models were fitted to the incidence data available from a country representative of each setting.
(PDF)

Figure S3 **Predicted vaccine efficacy for severe rotavirus gastroenteritis incidence in 0 to 4 year-olds.** Stepwise influence of improving the underlying natural history of protection, disease incidence and immunogenicity of vaccination.
(PDF)
