# Supplementary material for: Understanding Reduced Rotavirus Vaccine Efficacy in Low Socio-Economic Settings
Source: PLoS One. 2012 Aug 6;7(8):e41720. doi: 10.1371/journal.pone.0041720 (PMC3412858; doi:10.1371/journal.pone.0041720)
Supplement: Equations S1 — Model equations and additional description (PDF) [file pone.0041720.s001.pdf]

Model equations and additional description [Equations 1]

The model is a system of ordinary differential equations, the full model equations are:

$$\frac{dM_i}{dt} = -\mu M_i - \delta M_i + (1 - c_1 v_{1i})(1 - c_2 v_{2i})\delta M_{(i-1)} \text{ (when } i = 0, \delta M_{(i-1)} = \text{number of births)}$$

$$\frac{dS_{1i}}{dt} = \mu M_i - \lambda_i S_{1i} - \delta S_{1i} + (1 - cv_{1i})(1 - cv_{2i})\delta S_{1(i-1)}$$

$$\frac{dI_{ni}}{dt} = \lambda_i S_{ni} - \gamma I_{ni} - \delta I_{ni} + \delta I_{n(i-1)} \quad \text{(For } n = 1\ldots4)$$

$$\frac{dS_{2i}}{dt} = -\lambda_i S_{2i} + \alpha_1 \gamma I_{1i} - \delta S_{2i} + (1 - cv_{1i})(1 - cv_{2i})\delta S_{2(i-1)} + c(v_{1i} + v_{2i})\delta S_{1(i-1)} + c(v_{1i} + v_{2i})\delta M_{(i-1)}$$

$$\frac{dS_{3i}}{dt} = -\lambda_i S_{3i} + \alpha_2 \gamma I_{2i} - \delta S_{3i} + (cv_{1i} + cv_{2i})\delta S_{2i}$$

$$\frac{dS_{4i}}{dt} = \alpha_3 \gamma I_{3i} - \lambda_i S_{4i} - \delta S_{4i} + \delta S_{4(i-1)}$$

$$\frac{dR_i}{dt} = \sum_{n=1}^{3}(1 - \alpha_n)\gamma I_{ni} + \sum_{n=1}^{2}(1 - \alpha_n)(c_n v_{ni})\delta(M_{(i-1)} + S_{(i-1)}) + \gamma I_{4i} - \delta R_i + \delta R_{(i-1)}$$

where:
i = age group, defined as 1 month age groups from 0-59 months of age, then 5-24 year and 25-79 year age groups
M_i= those protected by maternal antibody in age group i
$S_{n,i}$ = susceptibles to nth rotavirus infection (n =1 to 4) in age group i
$I_{n,i}$ = infected by nth rotavirus infection (n=1 to 4) in age group i
R_i= recovered and immune to rotavirus infection in age group i
μ = rate of loss of maternal immunity
δ = rate at which individuals in age group i age into age group (i+1)
γ = rate of loss of infection
c = the proportion who seroconvert to dose 1 or 2 of vaccine
v_j= the proportion receiving dose 1 or 2 of vaccine where vaccination is given at age 2 and 4 months.
a_n = risk of becoming re-susceptible after nth rotavirus infection

λ_i= force of infection; rate at which susceptible individuals become infected in age group i is expressed as:

$$\lambda_i(t) = \sum_j qC_{ij} \sum_n I_{nj}(t)\xi_n$$

where C_{ij} is the number of physical contacts with individuals in age group j reported by individuals in age group i and $\sum_n I_{nj}(t)\xi_n$ represents the total number of symptomatic infectious individuals in age group j at nth infection at time t; ξ_n is the proportionof nth infections that are symptomatic.

Additional model description

An infectivity parameter (*q*) was estimated separately for each setting (described below) by fitting the model to incidence data using non-linear least squares in the software package Berkeley Madonna (Berkeley, CA, USA.). We used contact patterns measured for Great Britain[1] to represent age-specific mixing behavior across all settings, because similar studies assessing contact patterns have yet to be conducted in middle and low SES. Thus, *q* incorporates both the probability of infection given contact and a constant factor by which the mixing rate scales across the different settings (and can be greater than 1). All parameter values are shown in Table 1 of the main text. Seasonality and strain variation were not incorporated into the model.

1. Mossong J, Hens N, Jit M, Beutels P, Auranen K, et al. (2008) Social contacts and mixing patterns relevant to the spread of infectious diseases. PLoS Med 5: e74.
