# Supplementary material for: Understanding Reduced Rotavirus Vaccine Efficacy in Low Socio-Economic Settings
Source: PLoS One. 2012 Aug 6;7(8):e41720. doi: 10.1371/journal.pone.0041720 (PMC3412858; doi:10.1371/journal.pone.0041720)

Figure S1. Model structure. Figure (A) shows the original transmission model by Atchison et al [S1] and (B) illustrates how vaccination was included in the current analysis.

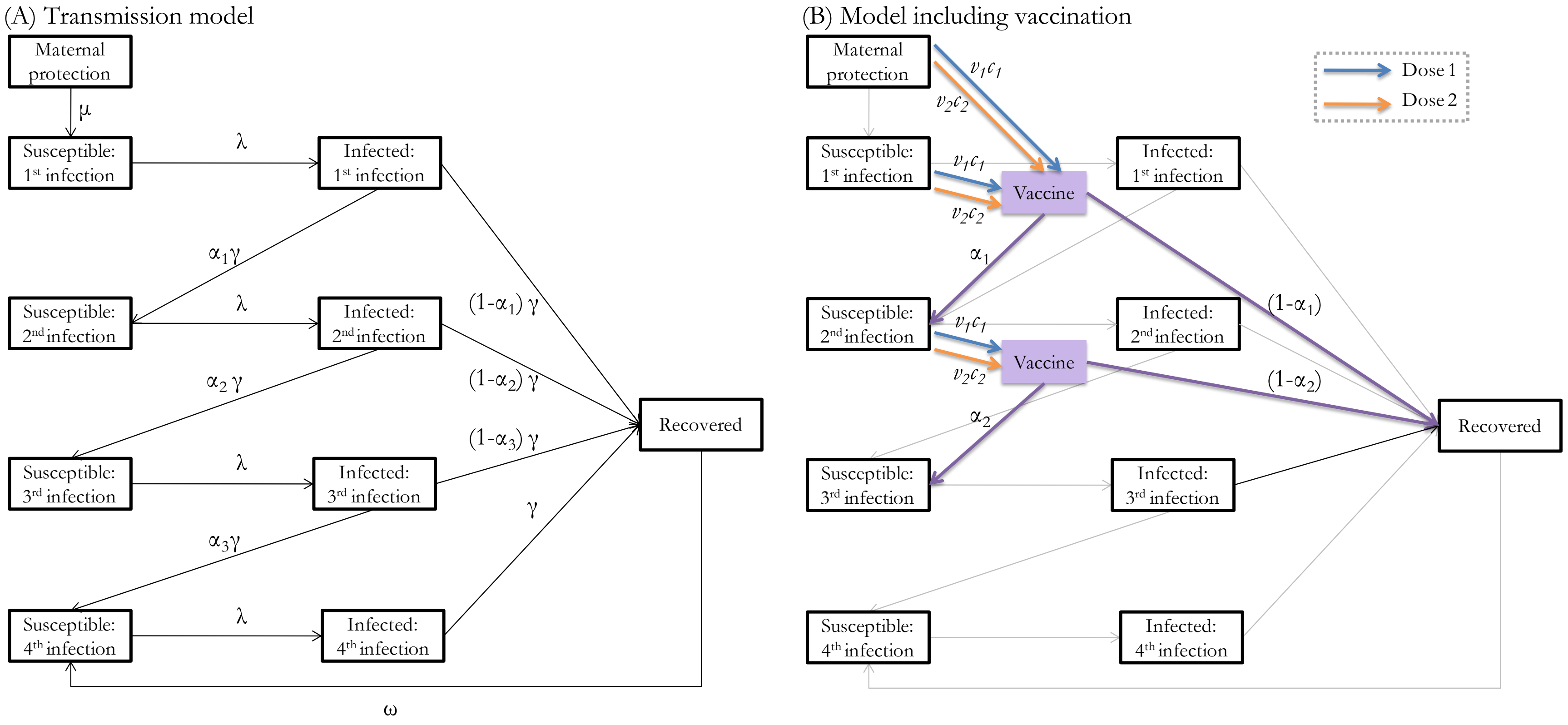

Supplement: Figure S1 — Model structure. Figure (A) shows the original transmission model by Atchison et al and (B) illustrates how vaccination was included in the current analysis. (PDF) [file pone.0041720.s002.pdf]
