# Supplementary material for: Understanding Reduced Rotavirus Vaccine Efficacy in Low Socio-Economic Settings
Source: PLoS One. 2012 Aug 6;7(8):e41720. doi: 10.1371/journal.pone.0041720 (PMC3412858; doi:10.1371/journal.pone.0041720)

Figure S2. Model-fitted and observed age-specific incidence of all RV-GE. Observed incidence rates (per 1000 child-years) are in shaded bars; fitted rates are shown in lines. Incidence data were not available across the age range from middle or low SES and in no settings was the age distribution available from the age-intervals of interest in this modeling study, so the models were fitted to the incidence data available from a country representative of each setting.

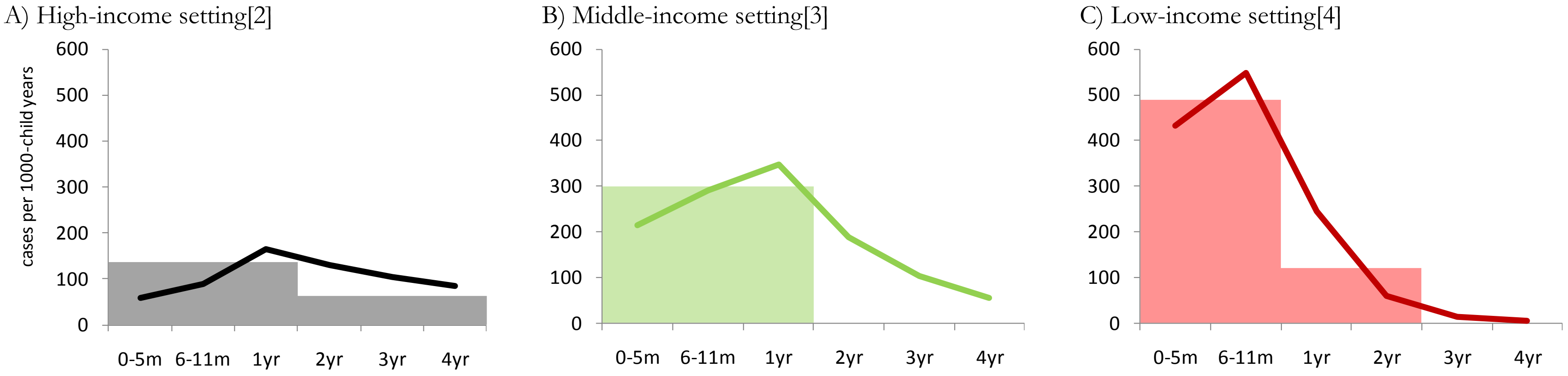

Supplement: Figure S2 — Model-fitted and observed age-specific incidence of all RV-GE. Observed incidence rates (per 1000 child-years) are in shaded bars; fitted rates are shown in lines. Incidence data were not available across the age range from middle or low SES and in no settings was the age distribution available from the age-intervals of interest in this modeling study, so the models were fitted to the incidence data available from a country representative of each setting. (PDF) [file pone.0041720.s003.pdf]
