# Supplementary material for: Understanding Reduced Rotavirus Vaccine Efficacy in Low Socio-Economic Settings
Source: PLoS One. 2012 Aug 6;7(8):e41720. doi: 10.1371/journal.pone.0041720 (PMC3412858; doi:10.1371/journal.pone.0041720)

Figure S3. Predicted vaccine efficacy for severe rotavirus gastroenteritis incidence in 0 to 4 year-olds. Stepwise influence of improving the underlying natural history of protection, disease incidence and immunogenicity of vaccination.

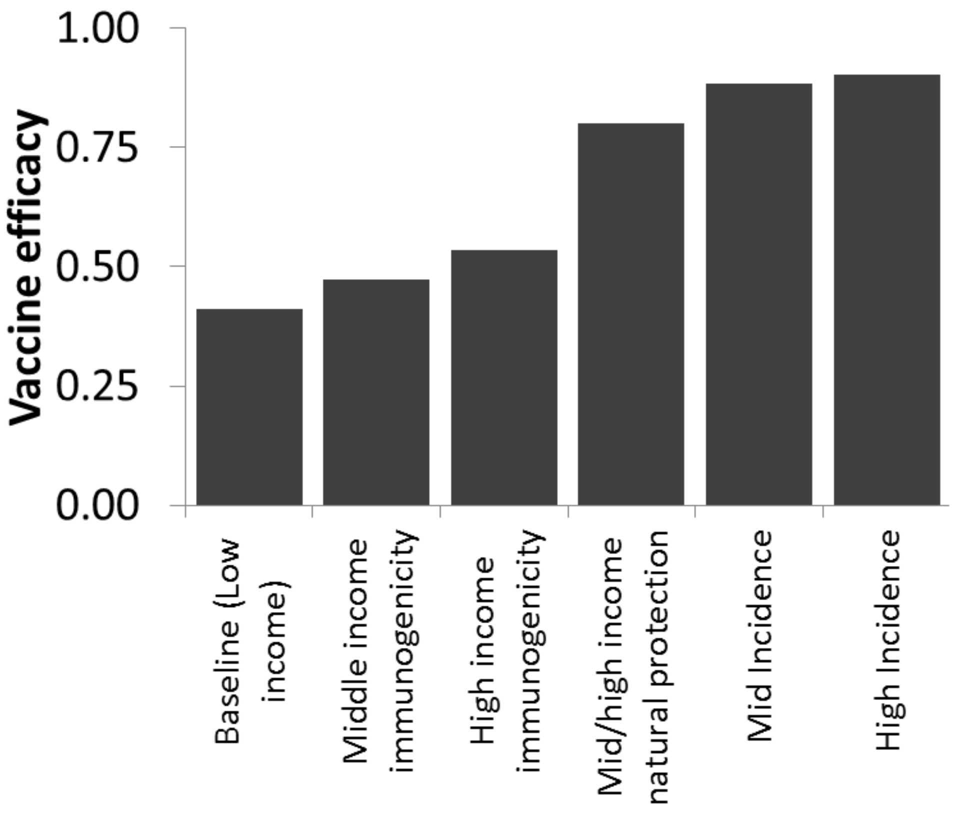

Supplement: Figure S3 — Predicted vaccine efficacy for severe rotavirus gastroenteritis incidence in 0 to 4 year-olds. Stepwise influence of improving the underlying natural history of protection, disease incidence and immunogenicity of vaccination. (PDF) [file pone.0041720.s004.pdf]
